# Exploring the Relationship between Green Space in a Neighbourhood and Cardiovascular Health in the Winter City of China: A Study Using a Health Survey for Harbin

**DOI:** 10.3390/ijerph17020513

**Published:** 2020-01-14

**Authors:** Hong Leng, Shuyuan Li, Shichun Yan, Xiuli An

**Affiliations:** 1 School of Architecture, Harbin Institute of Technology, Harbin 150001, China; lishuyuan@hit.edu.cn; 2 Key Laboratory of Cold Region Urban and Rural Human Settlement Environment Science and Technology, Ministry of Industry and Information Technology, Harbin 150001, China; 3 Heilongjiang Provincial Center for Disease Control and Prevention, Harbin 150001, China

**Keywords:** winter city, neighbourhood, green space, cardiovascular health, risk factors

## Abstract

A severely cold climate has a significant impact on cardiovascular health, involving temperature, air environment, exercise and diet. Existing studies have revealed that green space, as an important health resource, may play a positive role in promoting cardiovascular health through the air environment and exercise. Studies focusing on the correlation between green space and cardiovascular health are rarely carried out in winter cities. The purpose of this paper is to take a winter city in China as an empirical case to explore the correlation between green space in a neighbourhood and cardiovascular health in a representative sample at the neighbourhood level, combining the results with Urban Residential Area Planning and Design Standards (GB50180-2018) in China and the existing research. The results showed that green space characteristics of a neighbourhood were related to cardiovascular disease and some of its risk factors. In neighbourhoods with a Green Space Ratio lower than 28%, residents had a higher risk of physical inactivity, overweight or obesity, hypertension and stroke. In neighbourhoods with a Green View Index lower than 15%, residents had a higher risk of physical inactivity, overweight/obesity, hypertension, dyslipidemia and stroke. A correlation was found between evergreen tree configuration type and the prevalence of overweight/obesity and hypertension. No correlation was found between the type of sports field and cardiovascular disease and its risk factors, except for hypertension. Residents’ cardiovascular health scores also showed significant differences among neighbourhoods with different green space characteristics. Intervention efforts may benefit from emphasising the importance of improving the Green Space Ratio and Green View Index effectively in a neighbourhood to reduce the risk of cardiovascular disease.

## 1. Introduction

Climate is closely related to health. The significant impact climate has on public health should not be ignored. A large number of studies have found that cardiovascular disease morbidity and mortality are higher in high-latitude and severe-cold regions and in winter [1]. There is a U-shaped curve between air temperature and cardiovascular mortality—for every one-degree decrease in air temperature in severely cold regions, cardiovascular mortality increases by 1% [2]. Pressman N., one of the founders of the Winter Cities Association, proposes that a winter city is a city with a mean daily maximum air temperature of 0 °C for two months or more in a year [3]. In China, a winter city is usually defined as a city whose mean daily air temperature is below 0 °C for more than three months in a year, with the characteristics of low temperature, much ice and snow, and a long winter [4,5]. According to the International Association of Winter Cities, there are at least 30 countries located in the Northern Hemisphere, and more than 0.6 billion people experience winter [6]. According to the Sixth China Population Census, there were around 93.55 million people living in winter cities in 2010, accounting for 13.98% of the total population in China. How to overcome the influence of climate factors to promote public health in winter cities where cardiovascular health problems are more serious is a key point for the prevention and treatment of cardiovascular diseases with regards to the vast winter cities both in China and the world.

Compared with other countries with winter cities, standards of city size in China are relatively large; cities with a non-agricultural population of less than 200,000 in urban and suburban areas are defined as small cities [7]. In Finland and Denmark, residential areas with more than 200 to 250 people are identified as cities; the standard in the United States is 2500 people [5]. In terms of climate, with a typical continental monsoon climate, the air temperature is lower than that of cities at the same latitude. In terms of population density, a study showed that the population density is lager, with generally 10,000 people/km^2^, compared with 900–5000 people/km^2^ on average in other countries [5]. In terms of the air environment, due to a long heating period, air pollution is serious in winter cities in China [8]. In terms of the travel environment, the construction of a slow travel system and maintenance management in winter still need to be improved. Residents mainly rely on motor vehicles for travel rather than walking or cycling in winter, which is quite different from Finland, Sweden and Denmark, where there is a well-maintained cycling environment in winter [9], and Norway, with residents even integrating skiing into daily transportation and leisure [10]. In terms of the residential environment, residence buildings are mainly 4–6-storey, 7-storeys and above high-rise buildings, rather than 2–4-storey independent or row houses in Nordic Europe, Canada and the United States [5]. Meanwhile, due to the shortage of land and a large population base, the sunlight standard in neighbourhoods in winter cities is lower, causing the space between residence buildings to be compressed and green space to be occupied. In terms of indoor fitness facilities, it was reported that compared with the United States, the development of China’s fitness centres is relatively backward, serving young users aged about 30.7 years old and mainly for the middle and high-income class [11]. Many middle-aged and elderly people prefer outdoor activities based on habits, the desire to be close to nature and costs. Generally, there is still a certain gap regarding support of the urban environment for health in China’s winter cities.

Diet characteristics and living habits of residents in China’s winter cities are unique compared with people from other areas. They usually have a high-salt diet, partake in fewer outdoor activities and physical exercise and experience excessive fat metabolism, causing overweight or obesity. A sudden drop in temperature causes the blood vessels of the human body to contract easily, leading to convulsion, anoxia, increasing heart burden. Many key risk factors are associated with the winter city environment and residents’ lifestyle. At the same time, medical research points out the importance and necessity of proper outdoor exercise in winter for the prevention and rehabilitation of cardiovascular diseases [12]. Therefore, a severely cold climate has a double-effect on cardiovascular health.

A core component of social–ecological models published by the World Health Organization is that urban space is an important factor influencing public health through affecting human behaviour, which reveals that reduction of the risk of illness in a larger population may be realised by optimizing urban space [13]. The study on the burden of cardiovascular disease risk factors shows that the main risk factors of cardiovascular disease (CVD) are behaviour, metabolism and environmental factors. The sum of the three individual risk factors account for 20.4% of the total, and the sum of the interaction is 68.3%. However, by controlling these factors appropriately, the incidence of cardiovascular disease can be minimised [14]. Urban green space is considered as one of the key spatial elements [15]. According to the U.S. National Environmental Protection Agency, green space refers to land that is partly or completely covered with grass, trees, shrubs or other vegetation, including parks, community gardens, schoolyards, playgrounds, public seating areas, public plazas and vacant lots, which can provide recreational areas for residents and help to enhance the beauty and environmental quality of neighbourhoods [16]. Attributed to impact mechanisms of reducing air pollution [17] and promoting physical activity [18], studies suggest that living close to the natural environment is associated with long-term health benefits, including reduced death rates and reduced cardiovascular disease. A review identifying 12 studies across North America, Europe and Oceania found that the majority of studies shows a reduction of the risk of cardiovascular disease (CVD) mortality in areas with higher residential greenness [19]. A study observes an inverse correlation between the amount of green space and stroke incidence [20]. Relative studies also found that residential proximity to green space is associated with lower levels of diabetes, stroke and CVD [21], and higher rates of survival after stroke [22]. Results from a review in Netherlands found that higher residential greenness was associated with lower odds of coronary heart disease [23]. A prospective survival analysis in Canada found that higher levels of greenness were associated with a lower risk of CVD, ischemic heart disease and stroke mortality [24]. Additionally, results from a study of 15 American states showed that an increase in cardiovascular and respiratory illness was linked to tree loss [25]. Xiao et al. found that participants were more likely to have ideal cardiovascular health (CVH) rather than poor CVH in neighbourhoods that offered physical activity resources (19%), or a walking/physical activity environment (20%) [26]. The relationship between urban green space and cardiovascular health has been revealed by a number of studies. 

However, studies on the relationship between green space and health in different geographical environments and different spatial scales show different conclusions. For example, a study pointed out that no health effect was found at the urban level, and there was no significant difference in residents’ health risk factors between the cities with the highest and lowest green space coverage [27]. The health effect of green space at the micro-neighbourhood level may not be reflected at the overall level of the city [28]. For another example, a cross-sectional study in Kaunas failed to find a correlation between green space accessibility and cardiovascular risk factors [29]. Therefore, the applicability of the research conclusions in this field still needs to be further explored in different geographical environments and spatial scales. 

When obtaining green space characteristics as the independent variable of the study model, only a few studies [30] have combined the green space characteristics with national standards. The national standards are the most powerful and effective way to guide urban planning; whether the standards of green space are related to health benefits still needs to be studied. In addition, most studies focus on green space characteristics in the plane (e.g., accessibility, use, amount, type of green space, etc.), while those of three-dimensional space have been, traditionally, less studied. The Green Vision Index refers to the ratio of natural landscape, vegetation and other green scenes in the field of vision of people, which was less studied by European, American and Chinese scholars, but more studied by Japanese scholars [31]. In Japan, the concept was put forward in 1987 [32] and was included in the conventional indicators of urban green space evaluation in 2004 [33]. Xiao et al. proposed that the Green Vision Index can be used as an evaluation index of the amount of urban space from psychological perception and a compensation index of urban space development policy; in the course of promoting the construction of a green and healthy neighbourhood, it is necessary and possible to introduce the Green Vision Index as an evaluation index [32]. Wu et al. pointed out that when the Green Vision Index is more than 15%, natural feelings can be significantly increased, which may play a positive role in longevity based on visual psychology [34]. It is necessary to explore the relationship between the three-dimensional green space characteristic and health extensively.

In China’s winter cities, as the most important spatial element to carry the daily communication and exercise activities in the high-density human settlements, green space in a neighbourhood is extremely valuable to health. Focusing on the cardiovascular health of residents in the context of a severely cold climate, the purpose of this study is to explore the correlation between green space and cardiovascular health among residents in the winter city of China at the micro-neighbourhood level, combining these micro-scale green space characteristics with relevant standards in Chinese residential planning and existing research results. Green space in the study refers to green space within the scale of a neighbourhood. In China’s Urban Residential Area Planning and Design Standard (GB50180-2018) [35], a neighbourhood is defined as the area where residence buildings are relatively concentrated in the city. Green space is defined as green space built for a neighbourhood—including parks, community gardens, schoolyards, playgrounds, public seating areas, public plazas, vacant lots—available for residents to have a rest or partake in sports activities. The index selected from the planning standard is the Green Space Ratio, which refers to the ratio of the total area of green space to the area of the neighbourhood. In China’s Urban Planning-Making Method, it is defined as a mandatory indicator in the Regulatory Detailed Planning (an important planning between the Master Planning at the strategic level and the Detailed Planning at the operational level), which is the only indicator that can reflect the ecological benefits [36]. In addition, the Green View Index representing the three-dimensional green space characteristic, evergreen tree configuration type, representing green space characteristic in winter and sports field type, representing sports facilities, were also included in the study to jointly represent the characteristics of green space.

It should be pointed out that in winter, compared with non-winter cities, not only the spatial characteristics of green space in winter cities are different (e.g., from green space to white space), but also the residents’ behavioural activity [37], which is an important mediating factor [18] in the relationship between green space and cardiovascular health, is greatly affected (e.g., with the outdoor comfort reduced, it becomes more difficult to walk to green space, and the total outdoor activity time of residents is shorter [38] and the frequency is lower [39]). Thus, the health benefits of green space in winter cities through promoting physical activity may be weakened by the climate. The differences of green space characteristics and residents’ behavioural activity (rather than only the spatial characteristics) make the relationship between green space and cardiovascular health in winter cities differ from that in non-winter cities, which also makes the research in winter cities specific and worth doing.

## 2. Materials and Methods

### 2.1. Context

Harbin is located at 45° latitude N and 128° longitude E and is known as a typical winter city in the northeast of China, the annual average temperature of which is 3.6 °C. Harbin is also the political, economic and cultural centre of Northeastern China. Medical research from Harbin shows that when the temperature in the deep winter is below −19 °C (an inflection point), the death toll in Harbin increases significantly with the decrease of temperature [2]. Cold temperatures are responsible for 2.7 per cent of deaths, especially for coronary heart disease patients who are the most affected by cold stimulation [2]. At the same time, Harbin has entered a fast development period of population aging. At the end of 2015, there were 1.85 million elderly people aged 60 years and above, accounting for 19.2 per cent of the total population in the city [40]. With a large number of elderly people at risk, the city is facing a very serious level of cardiovascular disease risk.

The neighbourhoods in Harbin are currently polarised. The neighbourhoods built in recent years usually have better environmental quality and infrastructure. However, more than 50% of neighbourhoods were built before the 1980s–1990s, where environmental quality and infrastructure were poor. They are distributed in the main districts of the city, where the majority of residence buildings are multi-storey and high-density. To resist the severely cold climate, the layout of the buildings is mainly enclosed, and some of them are of a determinant or hybrid layout. The scale of green space in the enclosed neighbourhoods is usually small and the activities are relatively monotonous [41]. Due to the influence of the severely cold climate on activity safety and comfort, the attraction of outdoor activities is weak and the space utilization rate is limited.

### 2.2. Study Cohort

“Monitoring project of chronic diseases and the risk factors of residents in Heilongjiang Province” is a large-scale investigation conducted by the Center for Chronic Disease Control and Prevention in Heilongjiang Province to master the epidemic situation and change trend of major chronic diseases such as hypertension and diabetes. According to the balance of spatial distribution and the existing working basis and conditions, the monitoring sites were selected in Harbin administrative regions and other cities and counties in Heilongjiang Province [42]. The recruited respondents were received by doctors in the hospitals at the monitoring sites, and the trained staff introduced the informed consent form to the respondents participating in the screening, and monitored the information after signing the form under the voluntary principle. Monitoring methods mainly included the questionnaire survey, medical physical examination, laboratory test, dietary survey, etc. [43]. The health survey data used in this study is from one of the components, which belongs to an administrative region of Harbin, in a series of surveys carried out from 2013 to 2015. There were 5342 participants aged 20–98 in this monitoring site database. The research included: sociodemographic information, lifestyle information, health status information, results of screening stroke risk classification and home address information. The original database of 2013–2015 was exported from the original report database, and the participants with missing data were eliminated. The final database was formed by using Excel software offline, including 4155 samples of complete data. These samples were distributed in 12 neighbourhoods with multistory residence buildings of an administrative district in Harbin. They accounted for 6.1‰ of the total population in the administrative district, and 1.2‰ of the total population in all six main administrative districts of the city.

### 2.3. Measures

#### 2.3.1. Data Collection

The data in this study were collected on two levels. The individual (first) level included information on demographics, physical activity, height and weight, blood pressure, blood glucose and blood lipid. The contextual (second) level included information on the Green Space Ratio, Green View Index and sports field type in green space in a neighbourhood. These two levels were linked by neighbourhood for data analysis.

#### 2.3.2. Individual Level Variables

Age, sex, educational level, cardiovascular family history, smoking and physical activity levels were self-reported. Educational attainment was categorised into five groups: (1) primary school and below, (2) junior high school, (3) high school, (4) universities and colleges, (5) Master and above. Cardiovascular family history was categorised into two groups: (1) with a family history of cardiovascular disease, (2) without a family history of cardiovascular disease. Smoking was categorised into two groups: (1) current smokers or quit smoking for less than 12 months, (2) never smoked or successfully quit smoking for 12 months. The physical activity level was categorised into two groups: (1) physical inactivity, which means little or no physical exercise, (2) physical activity, which means exercising regularly.

The cardiovascular risk factors were physical inactivity, overweight/obesity, hypertension, diabetes and dyslipidemia. Height and weight were objectively measured and used to calculate the body mass index (BMI) as weight in kilograms divided by the square of height in meters. World Health Organization cutpoints for overweight/obesity were ≥25 kg/m^2^ for adults [43].

Blood pressure was measured by an electronic sphygmomanometer that had passed inspection by the quality inspection department and had been uniformly corrected before the on-site screening took place. The measurement unit was millimetres of mercury (mmHg) (1 mmHg = 1.33 kpa). The blood pressure measurement part was unified as the upper arm of the left hand. The first blood pressure measurement required the participants to rest quietly for at least 5 min. Two pressure measurements were made at least 1 min apart. According to the standard recommended by “China’s Prevention and Control of Hypertension Guidelines (2010)” [44], systolic blood pressure <140 mmHg and diastolic blood pressure <90 mmHg constitute normal blood pressure, and systolic blood pressure ≥ 140 mmHg or diastolic blood pressure ≥ 90 mmHg indicate hypertension.

In a fasting glucose test, fasting venous blood was taken during the physical examination after 12 hours of fasting. According to the diagnostic criteria of blood glucose in Chinese Guidelines for the Prevention and Treatment of Type 2 Diabetes: blood glucose < 6.1 mmol/L is normal blood glucose, blood glucose 6.1~7.0 mmol/L is impaired fasting blood glucose and blood glucose ≥7.0 mmol/L is diabetes [45].

Fasting venous blood after 12 hours of fasting was also used in the blood lipid test, to measure the level of biochemical indicators related to blood lipid, including total cholesterol (TC), triglyceride (TG), low-density lipoprotein cholesterol (LDL-C) and high-density lipoprotein cholesterol (HDL-C) [46]. According to the diagnostic criteria for dyslipidemia recommended by Guidelines for Prevention and Control of Blood Lipid in Adults in China, total cholesterol (TC) ≥ 6.22 mmol/L, triglyceride (TG) ≥ 2.26 mmol/L, low-density lipoprotein cholesterol (LDL-C) ≥ 4.14 mmol/L and high-density lipoprotein cholesterol (HDL-C) < 1.04 mmol/L were used as abnormal thresholds. Dyslipidemia is diagnosed in any one or more of the above four indicators [47,48].

Stroke risk was assessed by doctors based on a Stroke Score Card (Table A1). Cardiovascular health score is defined by the adjustment by Chinese medical researchers according to the ideal cardiovascular health score proposed by the American Heart Association (AHA). The definition is shown in Table 1. Since there were six cardiovascular health indicators in our study, the scores of cardiovascular health score were 0, 1, 2, 3, 4, 5 and 6 from low to high in order. A higher score means the better cardiovascular health status [49].

Physical inactivity, overweight/obesity, hypertension, diabetes and dyslipidemia were used to represent independent cardiovascular risk factors; stroke risk and cardiovascular health score were used to represent a comprehensive status of cardiovascular health.

#### 2.3.3. Green Space Variables

The extracted characteristics of green space include four indicators: Green Space Ratio, Green View Index, type of evergreen tree configuration and type of sports field. The data came from land use data and first-hand field surveys linked to each participant’s home address.

The Green Space Ratio is measured by the proportion of green space area within neighbourhood divided by the total land area of the neighbourhood. The green space area came from the primary data of the green space scale obtained through a field survey and ranging record in the research area. The total neighbourhood land area was derived from the data of land use status of Harbin in 2010 with the Google map of Harbin in 2015 as a reference. According to China’s Urban Residential Area Planning and Design Standard (GB50180-2018) [27], the Green Space Ratio should not be less than 28% in a neighbourhood with multistory residence buildings in severely cold regions (Climate Zone I), which was taken as the cutpoint of the Green Space Ratio in this study (Table A2). 

**Figure 1:**
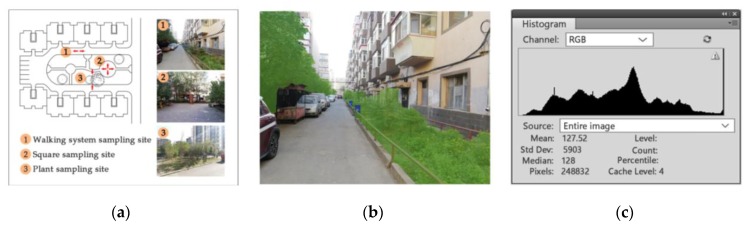
Measurement of the Green View Index. (**a**) Sample site setting and image collection in the field study; (**b**) green area extraction from images using an artificial visual interpretation method in Adobe Photoshop; (**c**) the Green Vison Index calculation based on Adobe Photoshop (source: combining the method of reference [35,51,52,53] with the images of neighbourhoods in the field study).

The Green View Index is a comprehensive value calculated by the average value of the walking system, open space and plants in a neighbourhood [35]. The walking system refers to the path system suitable for people and bicycle traffic in neighbourhoods, which has the functions of pedestrian traffic and pedestrian leisure, and can serve both non-motor vehicles and motor vehicles [50]. According to the principles of representativeness, universality and operability, the observation sampling sites of the walking system, open space and plants in each neighbourhood were selected. Figure 1 presents the main steps in measurement of the Green View Index. The observation sampling sites of the walking system were selected at the middle of the path, with pictures taken along the path. The observation sampling sites of plants were selected on the paths or squares where residents can easily view the green space landscape, in a certain distance from the plants, and pictures were taken towards plants. The observation sampling sites of squares were selected where the residents were concentrated, with pictures taken from squares towards the surrounding direction [35]. According to the spatial pattern of the green space, several survey observation images of the same size in different directions were collected from the perspective of humans in each sampling site. Two to four observation images were collected from one observation point, and then, these images were corrected according to the field of vision (80°–160° in the horizontal direction and 130° in the vertical direction), to ensure that the pictures can be included in the scope of people’s vision. Adobe Photoshop and the artificial visual interpretation method were used to extract the green plants (trees, grass, colour-leafed plants) in the observation image; the buildings, people, cars and other places were not calculated. The proportion of green plants in the image was calculated as the Green View Index of the sampling sites, and finally, the comprehensive Green View Index was calculated [35,51,52,53]. Based on the previous studies, 15% was taken as the cutpoint of the Green View Index. 

Statistics on evergreen tree configuration and sports fields were from the field survey and observation records. The existence of evergreen trees was taken as the classification basis of evergreen tree configuration types. The types of evergreen tree configuration were defined to be with evergreen trees, which means green space with one or more evergreen trees, and without evergreen trees, which means green space without any evergreen trees. The existence of exercise facilities was taken as the classification basis of sports field types. The types of sports fields were defined as active, which means venues with one or more physical activity facilities (exercise equipment, walking paths, courts), and passive, which means venues without any physical activity facilities.

### 2.4. Statistical Analysis

SPSS version 19.0 software for Windows (IBM Corp., Armonk, NY, USA) was used for statistical analysis. Significance was assumed at *p* < 0.05. Physical inactivity, overweight/obesity, hypertension, diabetes, dyslipidemia (five risk factors) and stroke risk were analysed independently, and cardiovascular health score was used in comparative analysis. 

Logistic regression models were calculated separately for cardiovascular risk factors and stoke risk with Green Space Ratio (first set of models), Green Vision Index (second set of models), evergreen tree configuration (third set of models) and sports field type (fourth set of models) as the independent variables. Dependent variables were included into the model using “without cardiovascular risk factor = 0, with cardiovascular risk factor = 1”, “without stroke risk = 0, with stroke risk = 1”, representing the cardiovascular health outcomes. Cardiovascular risk factors included physical inactivity, overweight/obesity, hypertension, diabetes and dyslipidemia. In order to identify the independent effects of three green space characteristics on five cardiovascular risk factors and one stroke risk, four sets of models (each set including six models) were constructed separately. In all models, age, gender and education level were adjusted. In the cardiovascular risk factor analysis models, smoking and cardiovascular family history were added to be adjusted as well. Each of the models was analysed using the Likelihood Ratio Test.

The results from the analysis were presented as odds ratios (OR) with 95% confidence intervals, which estimate the odds of being physically inactive, overweight/obese, suffering from hypertension, diabetes, dyslipidemia or stroke risk, representing the change of cardiovascular risk factors and stroke risk of respondents when the Green Space Ratio changed from more than 28% to less than 28%, Green Vision Index changed from more than 15% to less than 15%, evergreen tree configuration type changed from with evergreen trees to without evergreen trees and sports field type changed from active to passive.

A t-test of independent samples was used for the comparison between the cardiovascular health score of respondents living in neighbourhoods with different Green Space Ratio, Green Vision Index, evergreen tree configuration type and sports field type. 

## 3. Results

### 3.1. Participant Characteristics

A complete demographic and health profile was available for 5342 individuals. Complete information on residential address information could not be obtained for 1187 participants. A final sample of 4155 was available for analysis. The characteristics of the sample are presented in Table 2. There were slightly more male (52.3%) participants; the average age was 54.6 years old; the most-reported educational attainment level was primary school and below, about 63.9%. A total of 8.2% of respondents in this study reported a lack of physical activity. The mean cardiovascular health score was 5.60.

### 3.2. Green Space and Cardiovascular Risk Factors

According to land use information, field study and demographic characteristics, the neighbourhoods were similar to each other in terms of the location characteristic and surrounding built environment, and the social and economic characteristics of residents were also relatively consistent. From the binary logistic regression analyses on the association between the Green Space Ratio and cardiovascular risk factors (first set of models), we found a significant relationship between the Green Space Ratio and physical inactivity, overweight/obesity and hypertension, after adjustment for individual-level characteristics. The estimates are shown in Table 3 and can be interpreted as respondents in neighbourhoods with a Green Space Ratio lower than 28% are at higher risk of physical inactivity, overweight/obesity and hypertension, compared to those in neighbourhoods with a Green Space Ratio higher than 28%.

Likewise, the second set of models shows that neighbourhoods with a Green Space Ratio less than 15% were associated with a higher risk of physical inactivity, overweight/obesity, hypertension and dyslipidemia. The result was also after adjusted for individual characteristics. Participants living in neighbourhoods with a Green View Index of more than 15% had a lower risk of physical inactivity, overweight/obesity, hypertension and dyslipidemia.

In the third set of models, a significant relationship was detected between evergreen tree configuration type and being overweight/obese and hypertension. None of the models showed significant relationships between evergreen tree configuration type and cardiovascular risk factors except for overweight/obesity and hypertension.

In the fourth set of models, a significant relationship was detected between the sports field type and hypertension. None of the models showed significant relationships between the sports field type and cardiovascular risk factors except for hypertension.

### 3.3. Green Space and Stroke risk

The results of the logistic regression models are displayed in Table 4. It shows that a Green Space Ratio less than 28% and Green View Index less than 15% were associated with higher stroke risk, OR = 0.49 (0.37–0.68) and OR = 0.48 (0.38–0.61), compared to the reference “Green Space Ratio more than 28%” and “Green View Index more than 15%”, respectively. There was no significant relationship between evergreen tree configuration and stroke risk and sports field and stroke risk.

### 3.4. Green Space and Cardiovascular Health Score

The results of the comparison study of the cardiovascular health score among respondents living in neighbourhoods with different Green Space Ratio, Green View Index, evergreen tree configuration type and sports field type are shown in Table 5. There were significant differences in the cardiovascular health score corresponding to different characteristics of green space. Cardiovascular health score was higher among the residents in neighbourhoods with a Green Space Ratio higher than 28%, Green View Index higher than 15%, and active sports field. There was no significant difference in the cardiovascular health score among respondents living in different evergreen tree configuration types.

## 4. Discussion

Our research approach is based on the socio-ecological framework for the relationship between green space access and health by Lachowycz et al. [54], who proposed that physical health benefits, including weight management, lower blood pressure and reduced risk of conditions (such as heart disease and diabetes) are influenced by the characteristics of green space in the neighbourhood or workplace. Focusing on the green space in the neighbourhood, and taking the current national standards and the existing research into consideration, we selected the Green Space Ratio as the representation of the scale of the green space, Green View Index as the representation of the three dimensional composition of neighbourhood greening, evergreen tree configuration type as the representation of the green space characteristic in winter and sports field type as the representation of sports facilities. Cardiovascular risk factors and stroke risk were treated as dependent variables, with age, gender, education level and even smoking and cardiovascular family history adjusted.

The results of our study show that the Green Space Ratio and Green Vision Index of green space within one’s neighbourhood environment were associated with some cardiovascular risk factors and stroke risk. A Green Space Ratio equal to or lower than 28% is associated with physical inactivity, overweight or obesity, hypertension and stroke risk; a Green Vision Index equal to or lower than 15% was associated with physical inactivity, overweight or obesity, hypertension, dyslipidemia and stroke risk; green space without evergreen trees was associated with being overweight or obese and hypertension. However, the type of sports field was only found to be related to hypertension; it was not related to other cardiovascular risk factors or stroke risk. The results also showed that the cardiovascular health score among people in neighbourhoods with a Green Space Ratio higher than 28%, Green Vision Index higher than 15% or active sports field was statistically significantly higher compared to that among respondents in the neighbourhoods with a Green Space Ratio equal to or lower than 28%, Green Vision Index equal to or lower than 15% or a passive sports field. 

Our results are in accordance with other studies in which a more natural living environment is related to better health factors and lower risk factors, including objectively measured or self-reported health factors [55,56,57]. For example, studies found that there is a negative association between amount of green space, stroke incidence and cardiovascular health, respectively [58,59]. Another study reveals that greenness and the size of available public open space were inversely related to cardiometabolic risk [60]. A meta-analysis shows that more green space exposure is associated with a reducing incidence of stroke, hypertension, dyslipidemia and coronary heart disease: cardiovascular mortality −0.84 (95% CI 0.76, 0.93), diastolic blood pressure −1.97 (95% CI −3.45, −0.19), high-density lipoprotein cholesterol −0.03 (95% CI −0.05, <−0.01), and heart rate −2.57 (95% CI −4.30, −0.83) [61].

In terms of evergreen tree configuration, which partly represents green space characteristics in winter, this was found to be associated with overweight/obesity and hypertension. This may be due to the positive ecological effects of evergreen trees and their stress and attention recovery effects when a large number of deciduous plants wither in winter. In terms of the sports field, individuals living in neighbourhoods with better physical activity resources are associated with a lower prevalence of hypertension [62], a lower insulin resistance [63] and higher odds of having an ideal cardiovascular health score [64]. In our study, the sports field was mainly associated with hypertension, which was consistent with the above research conclusions. Medical research shows that promoting hypertension health plays an important role in the prevention of CVD. As one of the important and independent causes of cardiovascular diseases [65,66], it is of significance to maintain blood pressure health. Around the world, the high prevalence of hypertension is well reflected in the high prevalence of stroke and cardiovascular disease [67]. Therefore, green space with evergreen trees in winter and better sports fields may play a positive role in promoting health.

No association was found between sports fields and physical inactivity, overweight/obesity, diabetes, dyslipidemia or stroke risk. The discrepancy in results may, firstly, be affected by the type, content and place of physical activities. For example, some activities preferred by the middle-aged and elderly people (e.g., walking, taichi, stretching) are not completely dependent on sports facilities, and some people tend to choose indoor fitness facilities. Physical activities affected by external space may be weakened. Secondly, due to climate factors, outdoor physical activities are mainly suitable to be carried out in the daytime in transitional seasons and winter. During this period, the people who can carry out activities in green space in the neighbourhood without a time limit are mainly the elderly, while many office workers cannot use sports fields. With a lower temperature after work, the number of people who are willing to do outdoor activities is smaller. In this way, the health promotion effect of facilities on people’s activities may also be weakened. In addition, it may be related to the layout location of facilities. According to the field survey, during the same period in the morning, some facilities were in an area of sunshine, while some facilities were in an area in shadow. Most of the materials used in the exercise facilities are iron; thus, the facilities in the shadow area are difficult to use.

The challenge with green space in winter cities is that the physical-activity-promoting effect and ecological benefit of green space in winter could be weakened to a large extent. In terms of physical activity, under the influence of a severely cold climate, the residents’ overall activity participation rate in green space will decrease in winter [38]. On the one hand, the lack of outdoor activities in the long winter will not be conducive to human health in winter. On the other hand, long-term activities in low-temperature environments will have adverse effects on human cardiovascular health. Therefore, the existing research on winter exercise generally emphasises the appropriateness of outdoor activities in winter [68]. Meanwhile, a study on exercise characteristics of the elderly in winter cities shows that most of the elderly still adhere to a fitness regime in winter, and 80% of the elderly who participate in fixed fitness groups choose to adhere to daily outdoor fitness activities during winter, which shows that some residents still have strong needs to exercise in winter [69]. By providing the path and activity space, green space can play an important role for supporting physical activities [70]. In terms of ecological benefits, as a large number of deciduous plants wither in winter, it may be difficult to achieve dust retention and air purification fully. However, some studies have shown that the allocation of evergreen plants in winter and the adoption of appropriate planting methods (e.g., piece planting and row planting) can also play an effective role in reducing air pollution [71,72]. Therefore, although there exist challenges with green space in terms of the activity participation rate and ecological benefits, it can still play a positive role in promoting health. 

The advantage of this study is that it is a study based on a large-scale health survey of residents in China’s winter city; the first-hand investigation data is used in the analysis, which is combined with the requirements of the current national Urban Residential Area Planning and Design Standard (GB50180-2018) [27] for the green space in neighbourhoods in winter cities; and the Green Vision Index is added, which reflects residents’ perception of the environment and the three-dimensional composition of green space. It is a supplement to the existing research on green space and cardiovascular health. In addition, the research sample population and the scale of the green space were consistent. Because it was a single-city study, some comparative variables among different cities were not included, such as air quality. As the neighbourhoods were similar to each other, with relatively consistent location characteristics and coal-fired centralised heating, we assume that the health impact of air quality caused by these factors can be controlled, and the health value and health impact of green space characteristics in a neighbourhood are the main focus of the study. From the perspective of the impact of space, as the surrounding environment of the neighbourhoods where the respondents live were relatively similar, and their construction age was relatively close, the research results can be seen as a comparison of internal environmental factors in a neighbourhood to a large extent.

The disadvantage of this study is that the use of green space is not considered, and the use of green space by residents in neighbourhoods with relatively good green space resources may be more closely related to health outcomes [29]. Furthermore, the dichotomy method is used for data collection of physical activity, rather than the International Physical Activity Questionnaire or GPS in combination with accelerometer measurements, thus, the proportion of residents who can actually meet the WTO recommendations may be lower. In addition, not all of the residents’ physical activity occurred in green space. Apart from that, there may be elderly people who moved from the countryside with their children to the urban neighbourhood, that is, there may be migration. The local population may have stronger climate adaptability due to their extensive experience living in severely cold climate, and are more likely to maintain outdoor activities in winter. However, migrants may have a weaker ability to adapt to the climate and be unfamiliar with the new neighbourhood environment, thereby reducing their level of participation in outdoor activities. In addition, there is no exclusion of self-selection. In addition, in the process of analysis, diet was not adjusted, which plays an important role in the risk factors of cardiovascular diseases. People who prefer high salt diet often face higher risk of hypertension and cardiovascular diseases.

It should be noted that, under the influence of climate, the differences between winter cities and non-winter cities is reflected in both the spatial environment and residents’ activities [37]. Along with the change in green space characteristics caused by climate change (from green space to white space), the other important factor is the change in residents’ behavioural activity. Compared with non-winter cities, the overall activity duration and frequency of residents [38,39] in winter cities are more affected by winter. This activity change, which plays an important mediating role in the relationship between green space and cardiovascular health [18], makes the relationship between green space and cardiovascular health in winter cities different from that in non-winter cities. That is to say, the relationship between green space and cardiovascular health is special and meaningful in winter cities due to the changes of space and activity. Our research establishes a direct relationship between green space and cardiovascular health. The main characteristics we pay attention to are totality characteristics of green space. The spatial indicators with seasonal differences are not sufficiently comprehensive and prominent, and how an individual’s physical activity and its effect on cardiovascular health are affected by green space [73] in winter cities should be studied further. 

In future analysis, the identification of more comprehensive green space characteristics and the measurement of their health benefits in winter need to be further explored. The characteristics of green space discussed in this paper mainly reflect the totality characteristics, while the relationship between the detailed green space characteristics (e.g., plant layout, configuration method, quality, visual attraction) and cardiovascular health still need to be further studied. In addition, other important features of green space, such as the accessibility of sports fields in green space and the activity types that the sports fields can support also need to be explored more. With decreasing walking speed, tolerance time and travel distance [74,75], residents have higher accessibility demand for green space in winter. Accessibility is an important prerequisite for residents to visit green space in winter, and it is also an important factor that many scholars have paid attention to [76,77,78]. In terms of sports fields, climatic factors should be taken into consideration. In combination with the layout of sports fields during the suitable activity period, as well as people’s activity preferences, the matching of supply and demand should also be researched. 

Furthermore, research on the health benefits of the characteristics of green space in winter is scarce. Generally, the co-benefits of climate adaptation strategies have been scarcely investigated in terms of human health [79]. Therefore, future research on green space and cardiovascular health in winter cities should consider the identification of the unique characteristics of green space that may be correlated with cardiovascular health and the methods to quantify the health benefits of green space in winter. In addition, in terms of the impact mechanisms, study on the pathways of how green space influences CVD or its risk factors needs to be explored, for example, psychological factors can be added, and the mediating role of psychological factors and physical activity can be explored [61]. In addition, in terms of research design, as the cross-sectional study is less convincing, the results of this study need to be further validated by conducting a longitudinal study. Physical activity data, in this case, would be obtained in green space using the International Physical Activity Questionnaire or GPS in combination with accelerometers. 

## 5. Conclusions

The mechanism of cardiovascular disease in the population may involve individual characteristics, lifestyle, environment and other factors, but the neighbourhood environment plays an important role. The green space is an important spatial element in urban space, playing a key role in the reduction of air pollution and promoting physical activities among residents. Our study found some of the green space characteristics in the neighbourhood (Green Space Ratio ≥ 28%, Green View Index ≥ 15%) were related to better cardiovascular health and lower risk of CVD risk factors (physical inactivity, overweight/obesity, hypertension, stroke risk). A significant relationship was detected between green space without evergreen trees and being overweight/obese and suffering from hypertension. Except for hypertension, this study did not observe the association between the sports field type and other CVD risk factors and stroke risk. In future research, we should further identify more comprehensive green space characteristics and the measurement of their cardiovascular health benefits in winter, as well as the mediating roles of physical activity and psychological factors.

## Figures and Tables

**Table 1:** Definition of cardiovascular health score in our research.

Indicators	Definition of Cardiovascular Health Score in Our Research
Smoking	Never smoke or successfully quit smoking for 12 months
BMI	<25 kg/m^2^
Physical activity	Exercise regularly
Blood pressure	Systolic blood pressure < 140 mmHg and and diastolic blood pressure < 90 mmHg
Fasting blood glucose	<6.1 mmol/L
Total cholesterol	<6.22 mmol/L

**Table 2:** Demographic and health profile of the study participants in Harbin, China.

Characteristic	
Mean age, years (SD) ^1^	54.6 (10.3)
Proportion of men n (%)	2174 (52.3)
Education n (%)	
primary school and below	2654 (63.9)
junior high school	888 (21.4)
high school	547 (13.2)
universities and colleges	65 (1.6)
Master and above	1 (0.0)
CVD family history n (% yes)	205 (4.9)
Current smokers n (%)	225 (5.4)
Overweight/obesity (%)	727 (17.5)
Physical Activity n (% who met)	3816 (91.8)
Hypertension n (% yes)	200 (4.8)
Diabetes n (% yes)	53 (1.3)
Dyslipidemia n (% yes)	138 (3.3)
Stroke risk n (% yes)	430 (10.4)
Mean cardiovascular health score (SD)	5.60 (0.7)

^1^ SD-standard deviation.

**Table 3:** Logistic regression analysis of the odds of CVD risk factors by characteristics of green space.

Dependent Variables		Physical Inactivity ^1^	Overweight/Obesity ^2^	Hypertension ^3^	Diabetes ^4^	Dyslipidemia ^5^
Green Space Ratio	OR	**0.62**	**1.22**	**0.27**	0.79	0.68
	95% CI	**0.44, 0.87**	**1.01, 1.46**	**0.16, 0.47**	0.39, 1.61	0.42, 1.09
	*p*	**0.006**	**0.036**	**0.000**	0.515	0.108
Green Vision Index	OR	**0.53**	**1.28**	**0.31**	0.87	**0.61**
	95% CI	**0.39, 0.72**	**1.09, 1.52**	**0.20, 0.47**	0.47, 1.59	**0.41, 0.93**
	*p*	**0.000**	**0.004**	**0.000**	0.640	**0.021**
Evergreen tree configuration	OR	1.54	**1.44**	**0.41**	1.85	1.06
	95% CI	1.00, 2.36	**1.09, 1.91**	**0.19, 0.90**	0.77, 4.43	0.54, 2.11
	*p*	0.051	**0.010**	**0.026**	0.167	0.86
Sports field	OR	1.13	1.00	**0.68**	1.97	0.78
	95% CI	0.81, 1.57	0.82, 1.22	**0.48, 0.97**	0.90, 4.30	0.51, 1.18
	*p*	0.473	0.979	**0.022**	0.090	0.241

OR, odds ratio; 95% CI, 95% confidence interval; bolding indicates significance at *p* < 0.05. ^1^ Respondents with physical inactivity (= 1) compared to those exercise regularly (= 0). ^2^ Overweight/obese (= 1) compared to normal weight (= 0). ^3^ Respondents with hypertension (= 1) compared to those who were not suffering from it (= 0). ^4^ Respondents with diabetes (= 1) compared to those who were not suffering from it (= 0). ^5^ Respondents with dyslipidemia (= 1) compared to those who were not suffering from it (= 0).

**Table 4:** Logistic regression analysis of the odds of stroke risk by characteristics of green space.

Dependent Variables	Green Space Ratio			Green Vision Index			Evergreen Tree Configuration			Sports Field		
	OR	95% CI	*p*	OR	95% CI	*p*	OR	95% CI	*p*	OR	95% CI	*p*
Stroke risk ^1^	**0.49**	**0.37, 0.68**	**0.000**	**0.48**	**0.38, 0.61**	**0.000**	0.90	0.62, 1.32	0.599	0.81	0.64, 1.03	0.081

OR, odds ratio; 95% CI, 95% confidence interval; bolding indicates significance at *p* < 0.05. ^1^ Respondents with stroke risk (= 1) compared to those without it (= 0).

**Table 5:** Comparison of mean cardiovascular health scores of respondents by green space characteristics.

Green Space Characteristics		Mean Cardiovascular Health Scores	*T* Test (*p* < 0.05)	
			*t*	*p*
Green Space Ratio	>28% (n = 1029)	**5.68**	**4.875**	**0.000**
	≤28% (n = 3126)	**5.57**		
Green Vision Index	>15% (n = 1487)	**5.67**	**5.54**	**0.000**
	≤15% (n = 2668)	**5.55**		
Evergreen tree configuration	with (n = 323)	5.59	−0.02	0.984
	without (n = 3832)	5.60		
Sports field	active (n = 3278)	**5.63**	**5.887**	**0.000**
	passive (n = 877)	**5.47**		

Bolding indicates significance at *p* < 0.05.

## Appendix A

**Table A1:** Stroke risk score.

Eight Risk Factors (Applicable to People over 40 Years Old)			
Hypertension		□	≥140/90 mmHg
Blood lipid		□	Dyslipidemia or unknown
Diabetes		□	Yes
Atrial fibrillation		□	Irregular heartbeat
Smoking		□	Yes
Weight		□	Overweight/obesity
Physical activity		□	Lack of physical activity
Stroke family history		□	Yes
Assessment results	High risk	□	Three or more of these risk factors
		□	Previous history of stroke
		□	Previous history of transient ischemic attack
	Moderate risk	□	Being hypertension, diabetes or atrial fibrillation

A2 Regulations on the Green Space Ratio of neighbourhoods in Urban Residential Area Planning and Design Standard (GB50180-2018).

**Table A2:** Control index of Green Space Ratio of neighbourhoods in different building climate zonings.

Climate Zone ^1^	Category of Residence Building Layer Number	Minimum Green Space Ratio (%)
I, VII	Low-rise residence buildings (1~3)	25%
	Multistory residence buildings (4~6)	**28**%
II, VI	Low-rise residence buildings (1~3)	23%
	Multistory residence buildings (4~6)	28%
III, IV, V	Low-rise residence buildings (1~3)	20%
	Multistory residence buildings (4~6)	25%

^1^ China’s General Principles of Civil Building Design (GB50352-2005) divides China into seven main climate zones and 20 sub-climate zones. Winter cities located in Climate Zone I, which refers to severely cold regions are the study areas in this paper. Bolding indicates minimum Green Space Ratio in the neighborhood with multistory residence buildings in winter cities located in Climate Zone I.
